# Forecasting Dengue Hotspots Associated With Variation in Meteorological Parameters Using Regression and Time Series Models

**DOI:** 10.3389/fpubh.2021.798034

**Published:** 2021-11-26

**Authors:** Seema Patil, Sharnil Pandya

**Affiliations:** Symbiosis Institute of Technology, Symbiosis International (Deemed University), Pune, India

**Keywords:** dengue fever, climate change, machine learning, prediction, time series forecasting, regression model

## Abstract

For forecasting the spread of dengue, monitoring climate change and its effects specific to the disease is necessary. Dengue is one of the most rapidly spreading vector-borne infectious diseases. This paper proposes a forecasting model for predicting dengue incidences considering climatic variability across nine cities of Maharashtra state of India over 10 years. The work involves the collection of five climatic factors such as mean minimum temperature, mean maximum temperature, relative humidity, rainfall, and mean wind speed for 10 years. Monthly incidences of dengue for the same locations are also collected. Different regression models such as random forest regression, decision trees regression, support vector regress, multiple linear regression, elastic net regression, and polynomial regression are used. Time-series forecasting models such as holt's forecasting, autoregressive, Moving average, ARIMA, SARIMA, and Facebook prophet are implemented and compared to forecast the dengue outbreak accurately. The research shows that humidity and mean maximum temperature are the major climate factors and exhibit strong positive and negative correlation, respectively, with dengue incidences for all locations of Maharashtra state. Mean minimum temperature and rainfall are moderately positively correlated with dengue incidences. Mean wind speed is a less significant factor and is weakly negatively correlated with dengue incidences. Root mean square error (RMSE), mean absolute error (MAE), and R square error (*R*^2^) evaluation metrics are used to compare the performance of the prediction model. Random Forest Regression is the best-fit regression model for five out of nine cities, while Support Vector Regression is for two cities. Facebook Prophet Model is the best fit time series forecasting model for six out of nine cities. Based on the prediction, Mumbai, Thane, Nashik, and Pune are the high-risk regions, especially in August, September, and October. The findings exhibit an effective early warning system that would predict the outbreak of other infectious diseases. It will help the relevant authorities to take accurate preventive measures.

## Introduction

**Figure 1:**
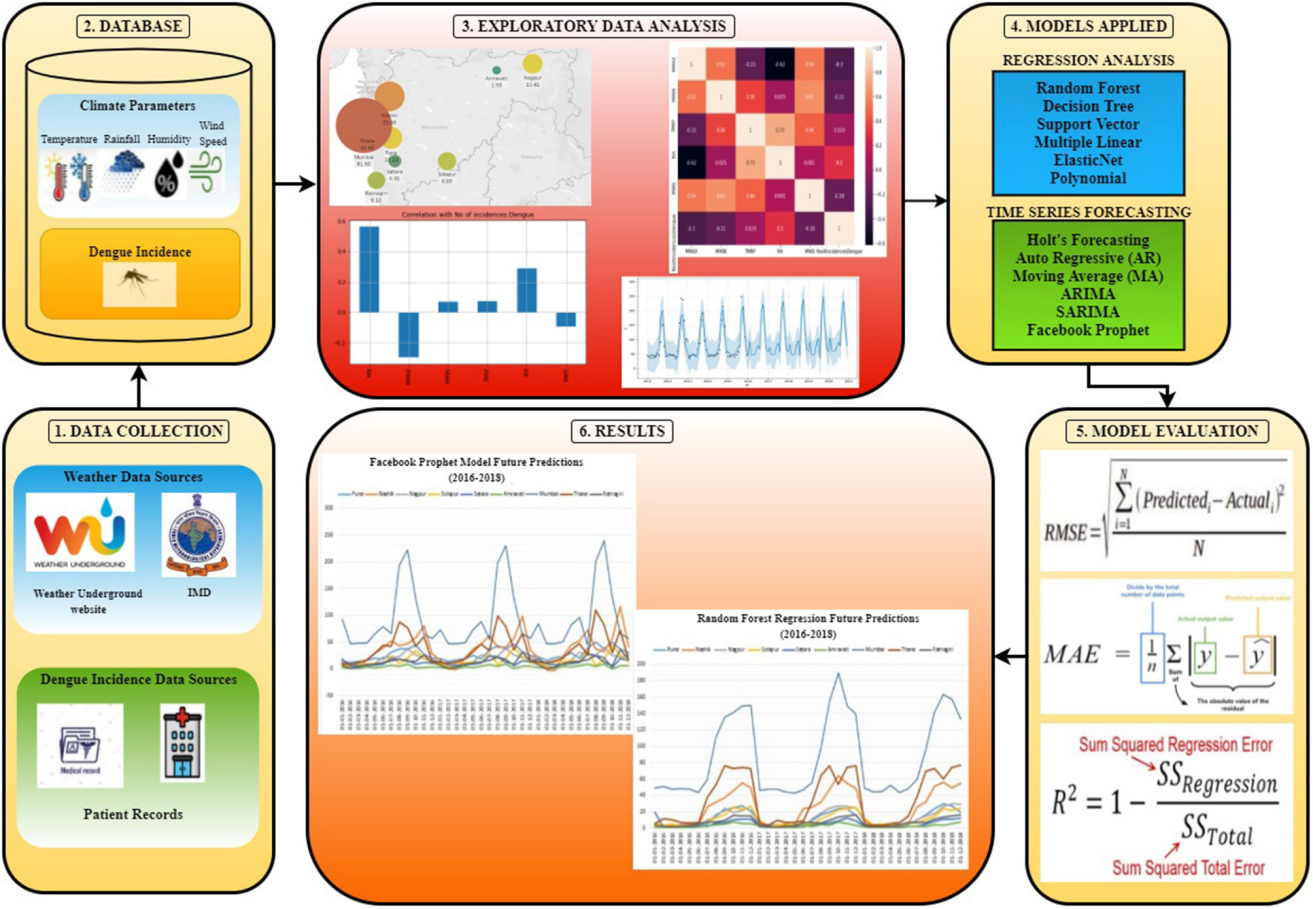
Effects of climate change on disease vectors.

Climate change is variations in climate variables such as temperature, humidity, precipitation, rainfall, wind speed, etc. Climate Change occurs due to natural activities such as variations in the sun, volcanic explosions, or human activities like the emission of carbon dioxide and other greenhouse gases that cause global warming. Infectious diseases are categorized into foodborne, airborne, waterborne, and vector-borne infectious diseases. Vector-borne infectious diseases are transmitted to humans by a microbe, called vectors, such as mosquitoes, ticks, flies, etc. Dengue is a vector-borne infectious disease carried by mosquito vectors that is most susceptible to meteorological conditions. According to WHO, this pandemic spreads over 128 countries across the globe and increased eight times over the last 20 years affecting 4.2 million people in the year 2020. Figure 1 shows the effects of climate change on disease vectors.

For understanding the spread of dengue, studying climate change and its effects specific to the disease is necessary. Temperature, rainfall, humidity, wind speed are the significant meteorological factors for the spread of dengue fever. Identifying the relationship between variation in these climatic factors and dengue incidences helps to predict the disease outbreak more accurately. An association has been found between the climatic parameters and dengue incidences for the selected locations in the proposed system. Machine Learning plays a vital role in developing a predictive model to understand the influx of dengue. Previously different classification and regression techniques were implemented for the prediction of dengue outbreaks for different locations. Considering varied geographic topography with changing climatic conditions and frequent disease outbreaks in the past, there is a need for better and accurate predictive models for early surveillance systems and improved prevention strategies. The following points highlight the paper's significant contributions:

- To collect climate and dengue incidence data for the selected locations for the past 10 years
- To identify the correlation between variations in climatic parameters and dengue incidences
- To implement various predictive models and show a comparative analysis based on different evaluation metrics
- To predict different climatic regions at risk in the future based on its climatic conditions

The following sections of the paper are organized as follows: Section Related Work describes related research work carried out for identifying the relationship between climate factors and vector-borne infectious diseases along with existing predictive modeling techniques available and its limitations. Section Proposed Work narrates the proposed system for dengue forecasting with variation in climate change. Section Methodology exhibits the methodologies used for forecasting dengue disease outbreaks. It includes different subsections such as data collection and integration, data preprocessing, exploratory data analysis, model execution, and evaluation metrics. Section Results and Discussion discusses details of predictive analysis and results. Finally, Section Conclusion, Limitations, and Future Work presents and concludes the author's research work.

## Related Work

Significant research has been carried out for understanding the association of meteorological variables with dengue incidences. This section describes the existing work related to the prediction of dengue incidences based on climatic factors using several machine learning techniques with its advantages and limitations.

Salim et al. (1) proposed a study to predict dengue outbreaks based on weekly dengue incidence data for the Selangor state of Malaysia. Several Machine Learning algorithms such as CART, ANN, SVM, and Naive Bayes create a predictive model. It has been found that the support vector machine model (SVM) best predicted dengue outbreaks. This research provides categorical output instead of continuous output. Liu et al. (2) implemented a unique approach for forecasting dengue incidences in Guangzhou, China. They integrated SVM-MLP machine learning approaches (3) with environmental features such as water collection sites, dustbins, etc. It performs better than models based on standard features (Temperature) alone. More standard features in addition to temperature and rainfall could be considered for better training of the ML Model. The SVR-based model Tanawi et al. (4) is proposed to predict dengue incidences in DKI, Jakarta. They concluded that SVR with a linear kernel provides better results than SVR with a radial kernel. Recently Mudele et al. (5) proposed a technique that uses a recurrent neural network (RNN) for forecasting the dengue mosquito vector population. This model is compared with random forest and k nearest neighbor for two Brazilian cities. They proposed that other deep learning models should be considered for the study (6–15). Mohapatra et. al. (16) investigated the effect of climate parameters on malaria outbreak using multilayer Perceptron and J48 classifier using WEKA tool. The results show that J48 is the most suitable model than MLP and has better accuracy and less error (RMSE). Also, temperature and humidity are more significant climate parameters than rainfall, and monsoon and post-monsoon are the peak periods for the outbreak. However, other factors such as demography, immunity within the population, society's socio-economic structure, availability of affordable public health facilities are not considered during the research (17). Cheng et al. (18) proposed distributed lag non-linear model to investigate the association between extreme weather events such as floods, heatwaves, high humidity, and dengue epidemic. The researcher implemented the model on daily dengue incidences and climate factors such as temperature, humidity, and rainfall for different cities of China. The threshold for each climate parameter is calculated, and risk for dengue outbreak is identified for the extreme weather events. The limitation of the research is that other time-variant factors such as changes in mosquito density, population movements and habits, and vector control measures are not considered for the study (19).

Xu et al. (20) analyzed dengue incidences data considering different meteorological factors. They proposed long short-term memory (LSTM) based recurrent neural network predictive model to predict monthly dengue cases using climate data for 20 Chinese cities. LSTM model shows the best performance for forecasting dengue incidences. But it is time-consuming compared to other models such as the backpropagation neural network and gradient boosting machine model. Appice et al. (22) formulated different strategies such as Auto Encoding, Window-based Data Slicing, and Cluster Analysis to discover temporal dynamics in temperature and dengue variables. They proposed a new multi-stage Machine Learning model called AutoTiC-NN (22) to find trend patterns between historical data of temperature and dengue in Mexico. The study proved that the model outperforms both in regression and time series forecasting analysis. Benedum et al. (23) compared machine learning, regression, and time-series models to forecast dengue cases and outbreaks in Peru, Puerto Rico, and Singapore. They concluded that Random Forest regression provided better results than Poisson Regression and ARIMA for short-term predictions while ARIMA was better for long-term forecasts. Nkiruka et al. (24) proposed a malaria incidence classification model (MIC) using climate parameters for six countries of Africa over 28 years. The research used k means clustering for outlier detection and the XGBoost model for classification. The proposed model is compared with other classification models such as ARIMA, SARIMA, SVM and showed the best results compared to other models.

Anno et al. (21) have integrated Spatiotemporal Hotspot analysis, RS Data, and a Machine Learning approach to develop a climate-based forecasting model to deliver early warning messages to the relevant public health authorities in Taiwan. This study uses two climate parameters (Rainfall and Temperature) to predict dengue outbreaks. Stolerman et al. (25) provide a better understanding of the long-term effects of climate conditions on the Aedes Aegypti (dengue causing mosquito) population. They have developed a new data-driven method using SVM algorithms to identify climate signatures that predict Dengue epidemics in Brazil. This research uses the binary threshold to classify epidemics/non-epidemics based on the Brazilian Ministry of Health. Two climate parameters (Frequency of precipitation and average Temperature) are used. Carvajal et al. highlighted the use of time lags of meteorological factors to predict dengue incidences. They concluded that Tree based Machine Learning methods (Random Forest, Gradient Boosting) performed better than conventional statistical techniques (GAM, SAIMAX) to predict a temporal pattern of Dengue incidences in Manila, Philippines. They also suggested that Relative Humidity is one of the most critical climate factors for their RF-LG model. All the variables are trained with keeping lag time in consideration to give an early outbreak prediction. Thus, this model cannot be used to predict an immediate output (17, 19, 26, 27). Despite continuous research, due to the varied topography of India, especially Maharashtra state having different climatic regions, there is a need to develop an accurate and enhanced predictive model for effective forecasting (2, 28–36). It will help the medical researcher and public health department promptly respond to the dengue outbreak and undertake corrective majors.

## Proposed Work

**Figure 2:**
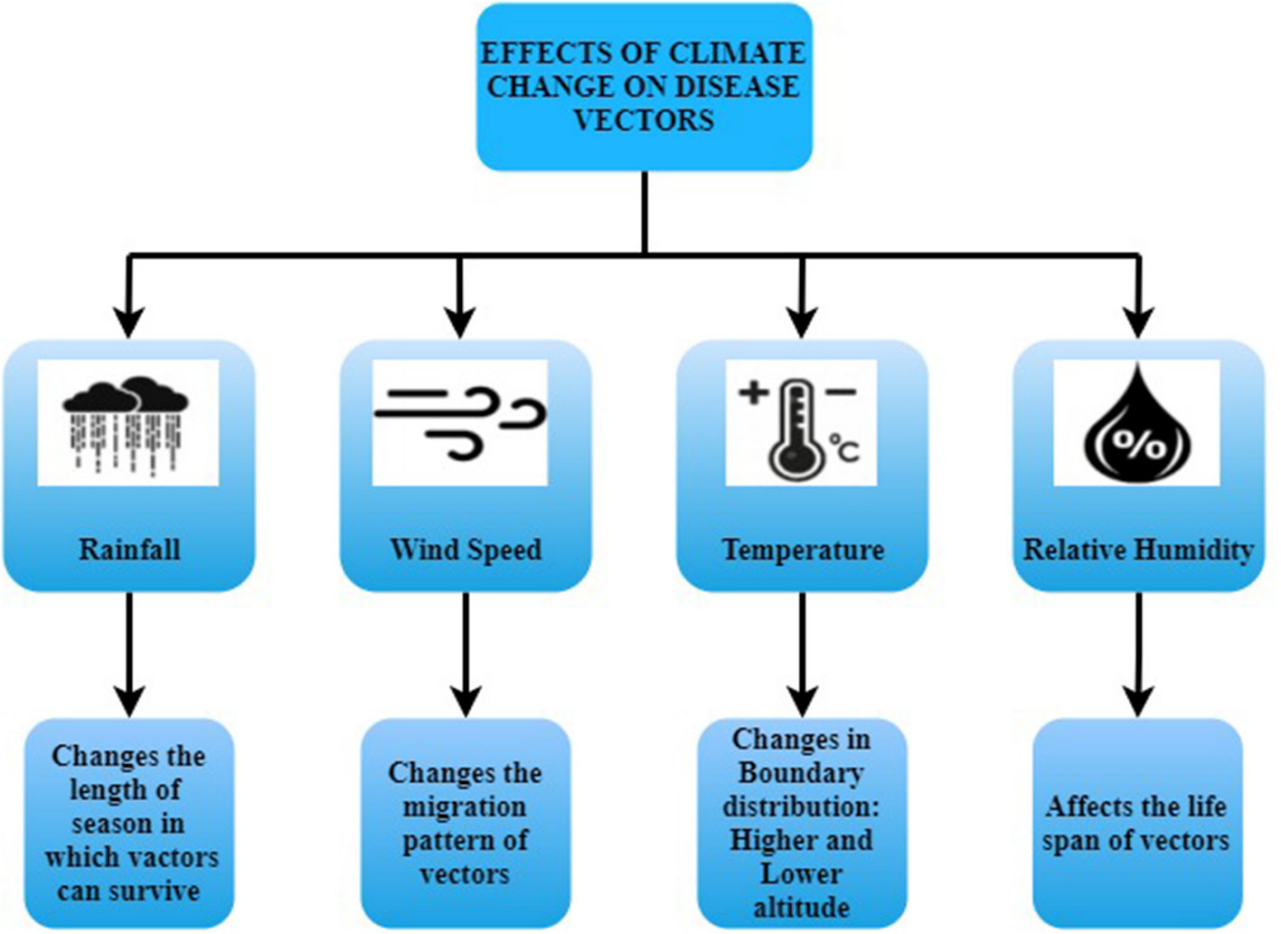
Schematic overview of the proposed system.

Figure 2 shows a schematic overview for dengue forecasting using regression and the time series model. It includes data sources and collection for both Climate parameters and dengue incidences. This is followed by data cleaning and integration in which missing data are imputed using the mean of the month data imputation technique. Exploratory data analysis is performed to find the correlation between climate parameters and dengue incidences. Feature engineering is carried out for feature selection and handling outliers. The impact of climate change includes indirect effects such as rising sea and temperature levels, extreme weather events such as droughts, floods, heatwaves, etc. The direct impact of climate change includes endurance, reproduction, or distribution of disease vectors which may affect human health. The climatic variations help in transmitting disease pathogens that may lead to infectious diseases (5, 16–19, 26, 27, 37–42).

Furthermore, the data is then split into training and testing data sets where training data is used to train different Machine Learning models–Regression Analysis and Time Series forecasting. These models are evaluated based on three evaluation metrics–Root Mean Square Error, Mean Absolute Error, and R Square Error. The models are compared to determine which models work best for different cities based on their geographic locations. Finally, locations at risk and outbreak period are predicted. Various visualization tools and techniques are used to represent the data and results effectively.

### Novelties and Contribution of the Proposed Work

The effect of the variation in climate factors with varied topography on infectious diseases such as dengue is an exciting research area. The proposed work illustrates the detailed analysis of the climate and health data for different locations of Maharashtra state of India. It includes finding a correlation between monthly climate factors such as mean minimum temperature, mean maximum temperature, mean wind speed, relative humidity, etc., with dengue incidences for different locations. These locations have diverse geographic topography and weather conditions. Based on the analysis, forecasting of dengue outbreaks is performed using time series and regression models. The performance of these models is compared using various evaluation metrics and identifies the best suitable models for the study. This research will help design an effective surveillance system that will accurately monitor and control the dengue outbreak in a timely manner.

### Methodology

**Figure 3:**
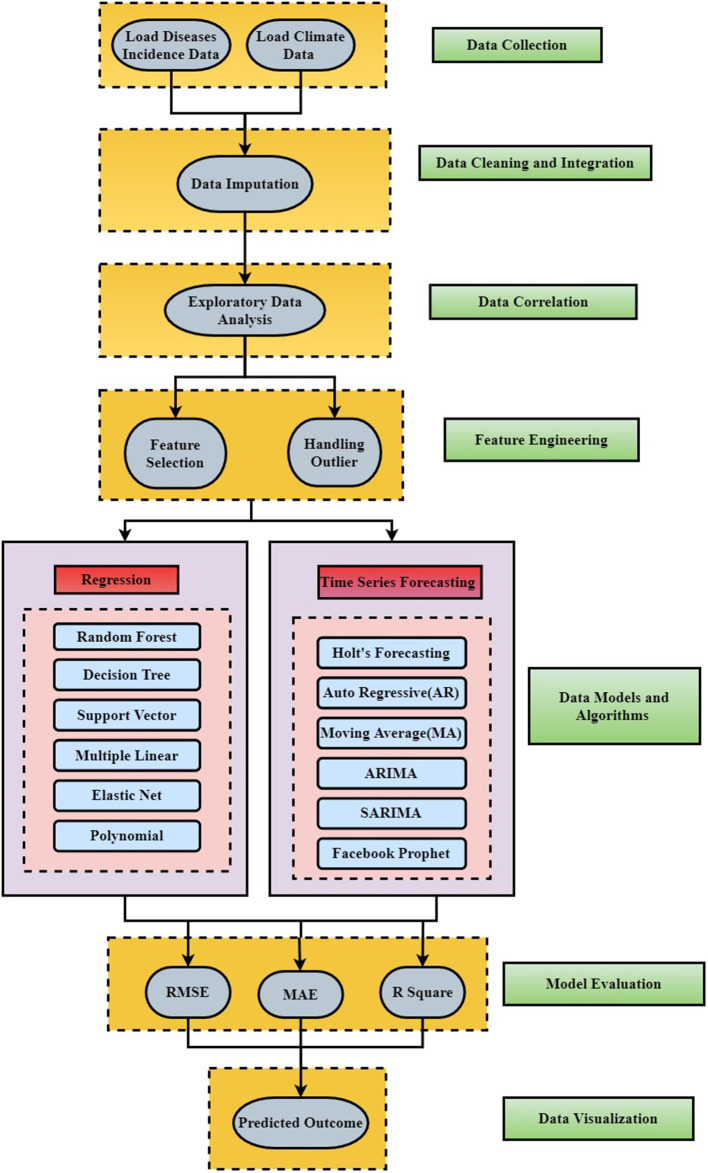
Summarized flow diagram for the forecasting model.

Figure 3 shows the detailed workflow and layered architecture for the construction of the dengue forecasting model. The following sub-section (Data Sources and Collection, Data Cleaning and Integration, Exploratory Data Analysis, Feature Engineering, Model Execution, Model Evaluation) elaborates different data sources and data collection process along with data preprocessing techniques implemented such as data imputation for missing values, climate and health data integration, feature selection, and outlier detection. Exploratory data analysis is performed to identify the correlation between climate parameters and dengue incidences using different visualization techniques such as heat maps, feature plots, etc. It also depicts different time series and regression machine learning models applied along with model evaluation metrics. Finally, dengue outbreak is forecasted for different cities of India for the next 3 years.

### Data Sources and Collection

#### Climate Data

Maharashtra has diverse climatic regions like Kokan, Khandesh, Desh, Vidarbha, and Marathwada. Based on the intensity of the disease and varied climatic conditions, nine cities like Mumbai, Thane, Ratnagiri, Pune, Solapur, Satara, Nashik, Nagpur, and Amaravati have been selected for the study. Monthly climate data is collected from Indian Meteorological Department (IMD) for 10 years from 2009 to 2019. The parameters in consideration are attributes such as Monthly Mean Maximum Temperature (MMAX) (°C), Monthly Mean Minimum Temperature (MIN) (°C), Total Monthly Rainfall (TMRF) (mm), Relative Humidity (RH) (%), and Mean Wind Speed (MWS) (km/h). Table 1 shows different region-wise locations of Maharashtra state along with population and climatic conditions.

**Table 1:** City wise population and weather conditions.

**Division name**	**City**	**Population (in Lakhs)**	**Weather conditions**
Konkan	Mumbai	125	Tropical, wet, and dry climate
Konkan	Thane	18.9	Tropical monsoon climate
Konkan	Ratnagiri	3.27	Tropical
Pune	Pune	31.2	Hot, semi-arid climate
Pune	Solapur	9.51	Dry, arid, and semi-arid climate
Pune	Satara	3.26	Tropical, pleasant climate
Nashik	Nashik	14.86	Mild tropical climate
Nagpur	Nagpur	24.1	Tropical Savanna climate
Amaravati	Amaravati	6.47	Tropical, wet, and dry climate
Aurangabad	Parbhani	3.07	Tropical, hot, and wet climate

#### Health Data

**Figure 4:**
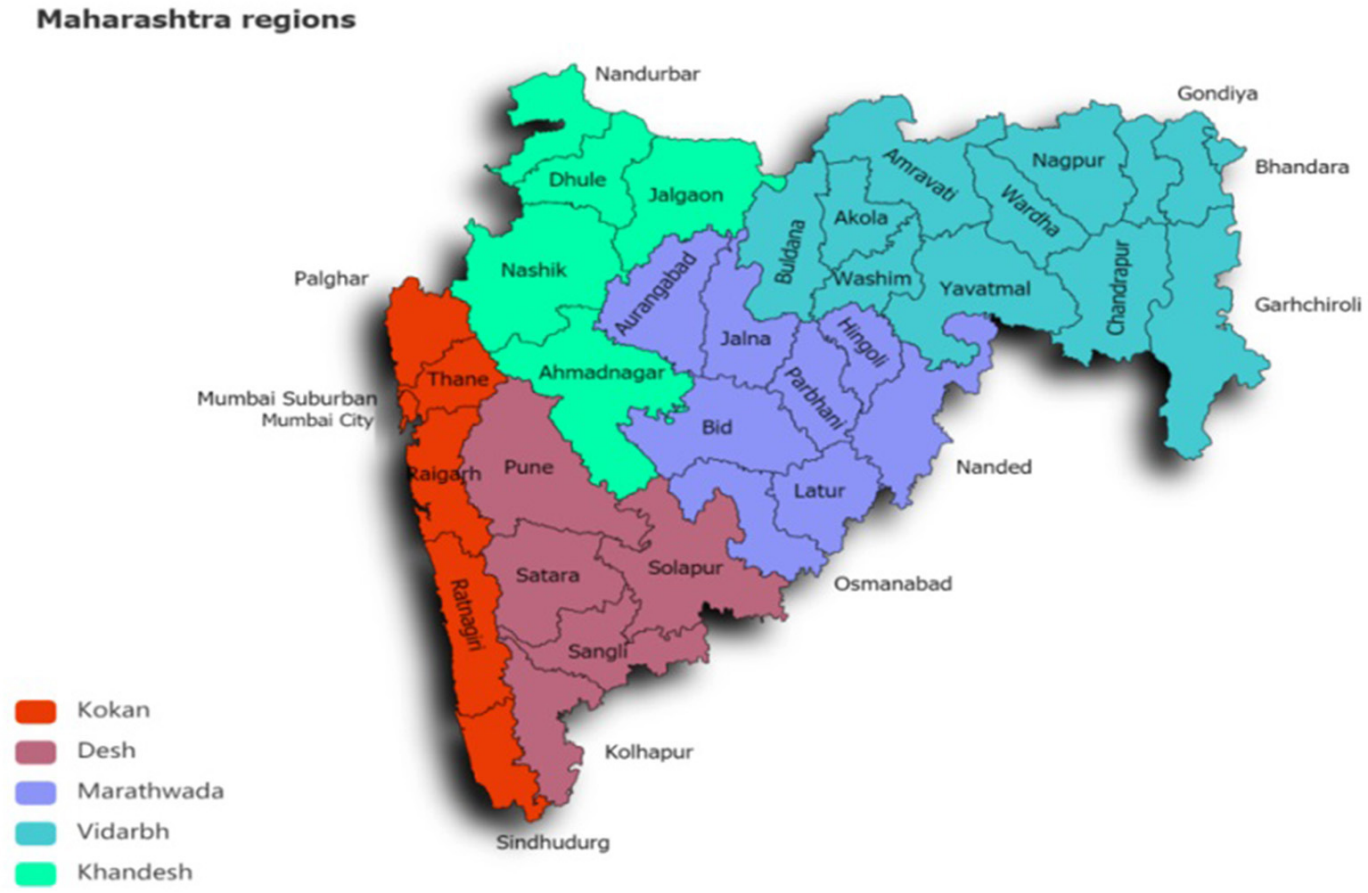
Map of Maharashtra state with region wise selected cities.

The monthly dengue disease incidence data is collected from the National Vector Borne Disease Control Program (NVBDCP) for targeted cities of Maharashtra state mentioned in the climate data section for 10 years from 2009 to 2019. The data collected is in excel format, having inconsistent and missing values. The climate and disease incidence data are integrated into the CSV file for all the nine targeted cities, and data preprocessing is performed. Figure 4 shows the map of Maharashtra state with region-wise selected cities for the study.

### Data Cleaning and Integration

To create the dataset, climate and dengue incidences data are collected and integrated. The dataset generated had inconsistent values due to the diverse nature of weather and health data. For each targeted city, a few irrelevant attributes are removed from the dataset during integration. The resulting dataset consists of missing values, especially in climate parameters. Data cleaning is performed to identify missing values. The data imputation technique is used to clean the dataset. The missing data were imputed using the mean of the Month Imputation technique. In this method, the missing values are replaced with an average of the previous values of the same month throughout different years. The mean of the month imputation function is given by:


(1) $$\begin{array}{l} \text{Vest}=\left(\sum \text{Tj}=1\text{Vij}\right)/\text{T} \end{array}$$

(2) $$\begin{array}{l} \text{Vest}=\left(\text{Vij}1+\text{Vij}2+\text{Vij}3+\ldots ..+\text{VijT}\right)/\text{T} \end{array}$$

The estimated value Vest for the missing attribute is calculated by the averaging sum of values (Vij) of the variable for the ith month of the year j, where T is the number of available data for that year. In the present study, the mean of Maximum temperature “MMAX” for August 2016 was missing in the given dataset from 2009 to 2019. The estimated value is calculated by an average of previous values of the same month throughout different years. This value was treated as a data point in place of the missing value.

### Exploratory Data Analysis

**Figure 5:**
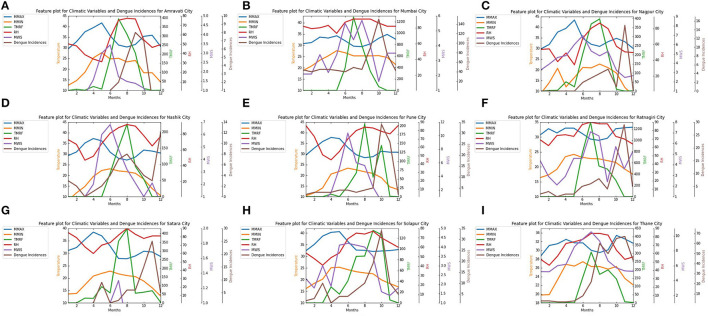
City Wise Features Plot for climate variables monthly mean minimum temperature, mean maximum temperature, Average rainfall, Relative humidity, Mean wind speed, and monthly dengue incidences for nine selected cities in Maharashtra from 2009 to 2019. **(A)** Amravati, **(B)** Mumbai, **(C)** Nagpur, **(D)** Nashik, **(E)** Pune, **(F)** Ratnagiri, **(G)** Satara, **(H)** Solapur, **(I)** Thane.

Once the dataset is cleaned, exploratory data analysis is performed to analyze attributes and summarize its characteristics using statistical techniques to discover useful patterns and graphical representation. City-wise feature graphs are plotted as shown in Figures 5A–I, and it is determined that each parameter for every city has a lot of variations, and there is no fixed pattern. So the correlation between each climate parameter and dengue incidences is found for all targeted locations to check which parameters are more significant.

Pearson correlation is performed on the dataset to determine the association between climate variables and dengue incidences, and heat maps are generated for each targeted city. Pearson correlation is a parametric test that measures the degree of relationship between two variables. It is the most suitable correlation technique based on the method of covariance and deals with numeric values. The person correlation function is given by Manogaran and Lopez (7):


(3) $$\begin{array}{l} r=\frac{\sum_{i=1}^{n}\left(ai-a\right)\left(bi-b\right)}{\sqrt{\left[\sum_{i=1}^{n}{\left(ai-\bar{a}\right)}^{2}\right]\left[\sum_{i=1}^{n}{\left(bi-\bar{b}\right)}^{2}\right]}} \end{array}$$

Here, the Pearson correlation coefficient function is employed to determine the relationship between the climate parameters and the number of dengue cases. Climate variables are monthly mean max temperature (MMAX), mean minimum temperature (MMIN), Rainfall (TMRF), Relative Humidity (RH), Mean Wind Speed (MWS).

Correlation between climate factors and dengue incidences shows that each climate variable affects the dengue incidences differently. The mean maximum temperature (MMAX) is negatively correlated with the incidences of dengue despite the locations. This implies that as the maximum temperature decreases, incidences of dengue have increased. Mean minimum temperature (MMIN) is weakly/moderately positively correlated with dengue incidences except for Nagpur. Relative Humidity (RH) is the primary climate factor and exhibits a strong positive correlation with dengue incidences for all locations of Maharashtra state. Similarly, total monthly rainfall (TMRF) is moderately positively correlated with incidences of dengue. As humidity or rainfall is increased, cases have shown an increase for all selected cities of Maharashtra. Maximum incidences occur between June to September, where the average rainfall is between 150 and 350 mm. Mean Wind speed (MWS) is a less significant climate factor and weakly negatively correlated with dengue incidences.

**Figure 6:**
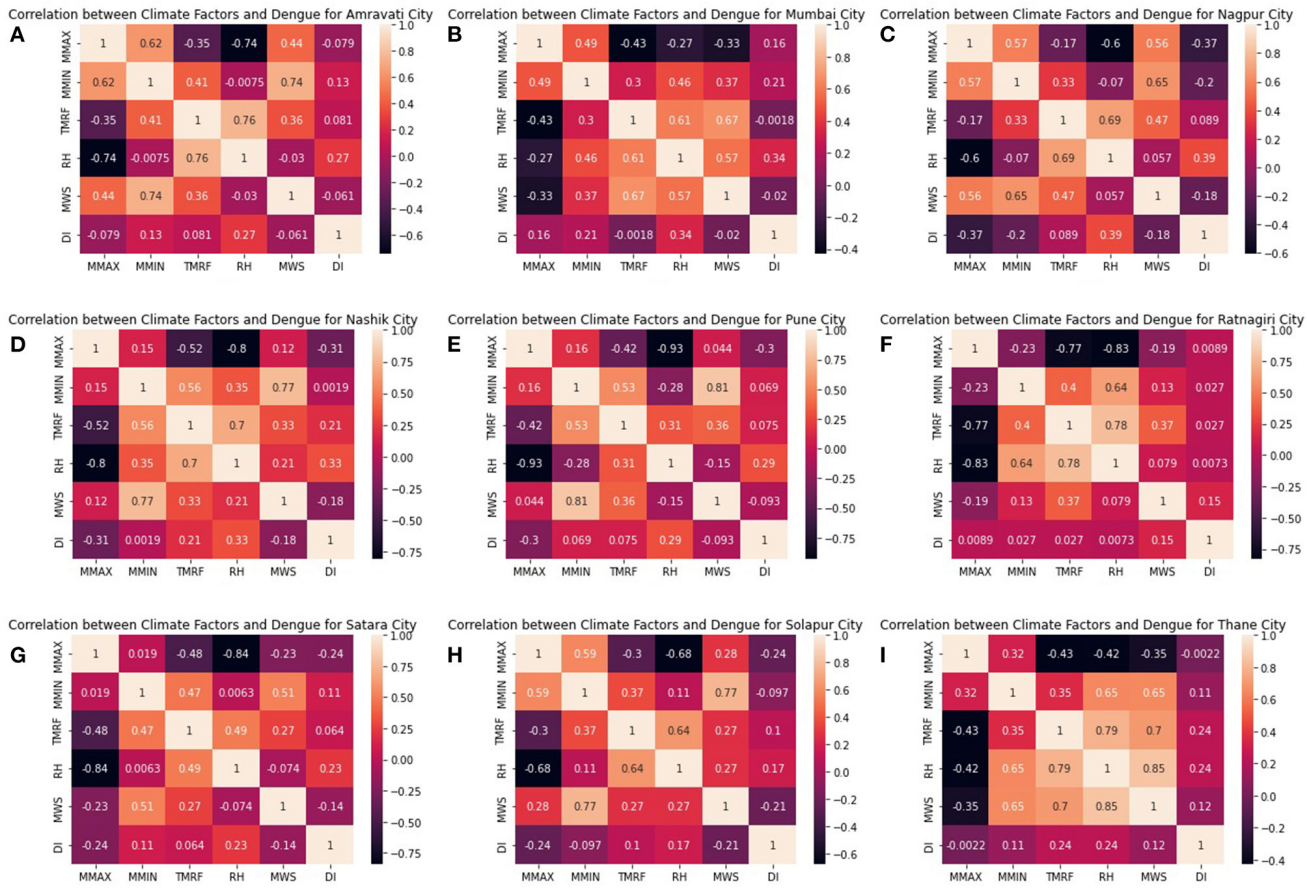
Correlation of monthly mean minimum temperature, mean maximum temperature, total monthly rainfall, relative humidity, mean wind speed climate parameters, and monthly dengue incidences: **(A)** Amravati, **(B)** Mumbai, **(C)** Nagpur, **(D)** Nashik, **(E)** Pune, **(F)** Ratnagiri, **(G)** Satara, **(H)** Solapur, **(I)**Thane.

Figure 6 shows city-wise graphs of the Pearson correlation of each climate parameter with the dengue incidences. These graphs are further used for feature selection based on results generated to identify the significant climates factors.

### Feature Engineering

The data quality is of utmost importance for developing a predictive model with better accuracy and faster performance. For this purpose, a few data preprocessing techniques are applied, such as outlier detection and feature selection, to improve the data quality. The meteorological data consist of extreme values for specific periods, such as extreme wind speed, rainfall, and humidity. Outliers in the dataset can reduce predictive modeling performance. So the final dataset was normalized to uniform into the same scale.

Feature or attribute selection is the process of selecting the most relevant attributes in a dataset that helps train the model faster, reduces overfitting, and improves the accuracy of the predictive model. Minimal redundancy maximum relevance feature selection technique is used for attribute selection on the dataset to select attributes with high correlation and low variance. For determining the relevant features, two measures are calculated: redundancy and relevance. The following equation is used to find the mean of logical values of each climate parameter for the selected city in terms of dengue incidences:


(4) $$\begin{array}{l} {\bar{c}}_{l}=\frac{1}{n_{i}}\sum_{k=1}^{n_{i}}C_{ik} \end{array}$$

Where, ${\bar{c}}_{i}$ : Means of climate parameter i, *n*_*i*_ : Number of climate parameters, *C*_*ik*_: the kth value of climate parameter i.

The below equation shows a variance of the climate parameters triggered by dengue incidences:


(5) $$\begin{array}{l} C_{i}^{2}=\frac{1}{n_{i}}\sum_{k=1}^{n_{i}}{\left(C_{ik}-{\bar{c}}_{l}\right)}^{2} \end{array}$$

The minimal redundancy condition for the redundancy measure can be expressed by:


(6) $$\begin{array}{l} \text{ min }R\left(D\right),\;R=\frac{1}{|d{|}^{2}}\sum_{yi;yj\in d}I\left(yi;yj\right) \end{array}$$

Where, min R (D) is the minimal redundancy for redundancy measure R, |*d*| is the number of features in the subset of feature D, and I (*yi*; *yj*) is the mutual information between feature i and j.

The maximal relevance condition for the relevance measure can be expressed by:


(7) $$\begin{array}{l} \text{ max }RL\left(D,a\right),\;RL=\frac{1}{\left|D\right|}\sum_{yi\in d}I\left(yi;a\right) \end{array}$$

Where max RL (D, a) is the maximal relevance for relevance measure RL and target activity a and I (yi; a) is the mutual information between the feature i and target activity a.

The smaller the value of the redundancy measure, the better the criteria for selection. Similarly, the higher the value of relevance measure, the better the feature selection. After exploratory data analysis is performed on the dataset considering several feature variables such as MMIN, MMAX, TMRF, RH, and MWS, different climate variables with high redundancy and low relevance are dropped for few cities under study, as shown in Table 2.

**Table 2:** Dropped variables.

**City**	**Dropped variables**
Amravati	N/A
Mumbai	TMRF
Nagpur	N/A
Nashik	MMIN
Pune	TMRF, MMIN
Ratnagiri	MMAX, RH
Satara	TMRF
Solapur	N/A
Thane	MMAX

### Model Execution

Regression is a supervised learning statistical method used to estimate the relationship between a dependent and one or more independent variables to determine trends in the data. It is used in the prediction of continuous values. The following regression models are implemented to predict dengue incidences across different cities based on climate parameters in the proposed system.

#### Support Vector Regression

It is the regression technique used to predict continuous ordered values. Some commonly used keywords in SVR are the kernel, hyperplane, boundary line, and support vectors. The primary purpose of SVR is to consider as many data points as possible within the boundary lines, and the hyperplane (best-fit line) must contain as many data points as possible. It is easy to implement and shows high prediction accuracy with excellent generalization capability. It can handle outliers very well.

#### Multiple Linear Regression

It is an extension of simple linear regression that models a linear relationship between more than one independent variable and a single dependent continuous variable. It is a technique for fitting a regression line through a multidimensional space of data points.

#### ElasticNet Regression

Elastic net is a type of regularized linear regression that includes two well-known penalties, the L1 and L2 penalty functions. The advantage of the elastic net model is that it permits a balance of both penalties, resulting in a more excellent performance on particular tasks than a model with either one or more penalties.

#### Polynomial Regression

Polynomial regression is a type of linear regression that estimates the connection as an nth degree polynomial. It is an example of multiple linear regression. Because Polynomial Regression is sensitive to outliers, the existence of one or two of them can have a negative impact on the results.

#### Decision Tree Regression

It is a regression model that breaks down a dataset into smaller subsets forming a tree with decision nodes and leaf nodes. Decision trees are very easy to visualize and reduce the uncertainty in the prediction. However, overfitting and underfitting are common problems with decision trees. If the hyperparameters are incorrectly set, the decision tree's output can vary dramatically.

#### Random Forest Regression

Random forest is the most commonly used machine learning technique that gives excellent results in predicting disease incidences based on climate conditions. It comprises many decision trees, each with the same node but different inputs, resulting in various leaves. It combines the results of the average of various decision trees. Overfitting can be avoided in the model by using Random Forest regression to create random subsets of the dataset.

Along with regression, the proposed system also used time series forecasting models to predict dengue incidences. Time series data is a sequence of different data points that measure a specific variable over an ordered period. In this method, time-series data extract meaningful statistics and other data characteristics to generate forecasts of our target variable. Different time series forecasting models are applied as given below:

**Holt's Forecasting:** It is time series forecasting method that depicts trends and seasonality from historical data. It is simple to implement and evolve with changing business requirements.

**Auto-Regressive (AR) and Moving Average (MA)**: Autoregression forecasting technique predicts future values using previous values in time series. It demonstrates linear relation between future and past values. It is used to forecast recurring patterns in the data. The moving average method uses an average of several past points to predict future points. In this method, short-term fluctuations and the effect of extreme values are reduced.

**Auto-Regressive Integrated Moving Average (ARIMA):** ARIMA model is a popular time series forecasting model which uses its lags to predict future values. It uses dependent relation between past observation and current observation. It involves subtracting recent observations from previous period observations several times. It is broken down into its subtypes to increase the accuracy of incidence predictions based on climate variability. This model does not support seasonal data.

**Seasonal Auto-Regressive Integrated Moving Average (SARIMA):** When seasonal components are added to the ARIMA model, then it is called SARIMA. It supports univariate time series data. Additional four seasonal elements in SARIMA are P, D, Q, and m, where P is seasonal autoregressive order, D is seasonal difference order, Q is moving average order, and m is the number of time steps (17).

#### Facebook Prophet Model

The Facebook prophet is a relatively new time series forecasting model developed in 2017 by the Facebook data science team as open-source software. In this model, irregular observations are permitted in the dataset as it ignores temporal data dependence. It is accurate, fast, and shows excellent performance as compared with other time series forecasting models. The prophet equation is given by:


(8) $$\begin{array}{l} x\left(t\right)=c\left(t\right)+s\left(t\right)+h\left(t\right)+u\left(t\right) \end{array}$$

Where x(t) is forecast value, c(t) is the trend, i.e., change over a long period, s(t) is the seasonality, h(t) is the effect of the holiday, u(t) is unconditional changes or error. This model works best with time series with substantial seasonal influences and historical data from multiple seasons. It is robust to outliers and handles missing values very well. This model gives the best performance for six out of nine cities to forecast dengue incidences based on climate variations in the proposed system.

### Model Evaluation

Once all regression and time series forecasting models are trained, the performance of the models is evaluated using three evaluation metrics: Root Mean Square Error (RMSE), Mean Absolute Error (MAE), and R Square Error (*R*^2^). RMSE is the Standard Deviation of predicted errors. Lower RMSE values indicate better models. RMSE is evaluated by the Equation (2):


(9) $$\begin{array}{l} \text{RMSE}=\sqrt{\sum_{i=1}^{\text{N}}{\left({\text{x}}_{\text{t}}-{\bar{\text{x}}}_{\text{t}}\right)}^{2}/\text{N}} \end{array}$$

Here, xt is actual dengue incidences for time t and ${\bar{x}}_{t}$ is the predicted number of incidences by the model.

Mean Absolute Error (MAE) is the difference between the actual values and the predicted values. Lower MAE values indicate better models. MAE is evaluated by Equation (1):


(10) $$\begin{array}{l} MAE=1/n\sum_{i=1}^{n}|{\text{X}}_{\text{i}}-\text{X}| \end{array}$$

R Square Error (*R*^2^) is also known as the coefficient of determination. It tells us how well a model fits on a dataset. It indicates how close the regression line is to the actual data. The *R*^2^ value closest to 1 is considered to be the best value. The equation given below evaluates the value of *R*^2^ (20):


(11) $$\begin{array}{l} R^{2}=1-\frac{{\text{SS}}_{\text{Regression}}}{{\text{SS}}_{\text{Total}}} \end{array}$$

## Results and Discussion

Based on the exploratory data analysis, it was observed that each climate variable affects the dengue incidences differently. The average temp range in Maharashtra state is between 26 and 43°C. As shown in Figures 6A–H, histogram graphs generated after performing Pearson's correlation shows that mean maximum temperature (MMAX) is negatively correlated with the incidences of dengue despite the locations. This implies that as the maximum temperature decreases, incidences of dengue have increased. Mean minimum temperature (MMIN) is weakly/moderately positively correlated with dengue incidences except for Nagpur. Relative Humidity (RH) is the primary climate factor and exhibits a strong positive correlation with dengue incidences for all locations of Maharashtra state. Similarly, total monthly rainfall (TMRF) is moderately positively correlated with incidences of dengue. As humidity or rainfall is increased, cases have shown an increase for all selected cities of Maharashtra. Maximum incidences occur between June and September, where the average rainfall is between 150 and 350 mm. Mean Wind speed (MWS) is a less significant climate factor and weakly negatively correlated with dengue incidences. Table 3 shows rudimentary observations for all five climatic parameters. The performance of all the regression and time series forecasting models for each city is evaluated and compared.

**Table 3:** Rudimentary observations.

**Climate parameters**	**Rudimentary observations**
Relative humidity	For RH > 55%, Number of incidences increases
Monthly maximum temperature	For temp 30°C < MMAX <35°C, Number of incidences increases
Monthly minimum temperature	For temp 18°C < MMIN <25°C, Number of incidences increases
Mean wind speed	For MWS <2.5 kmph, No of incidences increases
Total monthly rainfall	For rainfall, 150 mm < TMRF <250mm, No of incidences increases

Tables 4–**12** present city-wise performance comparison for all regression and time series forecasting models. The best fit values for each metric are highlighted in bold.

**Table 4:** Performance metrics comparison table for Amravati.

**Regression models**	**RMSE**	**MAE**	***R* ^**2**^**	**Time series forecasting**	**RMSE**	**MAE**	***R* ^**2**^**
Random forest	5.22	3.43	−0.32	Holt's Forecasting	2.22	2.09	−0.39
Decision tree	5.35	2.88	−0.36	AR	1.95	1.41	−0.07
Support vector	4.82	**2.11**	−0.12	MA	**1.93**	**1.29**	−0.05
Multiple linear	4.52	3.12	0.01	ARIMA	2.19	1.39	−0.35
ElasticNet	**4.49**	3.05	**0.02**	SARIMA	1.98	1.92	−0.11
Polynomial	7.44	5.7	−1.68	Facebook Prophet	3.14	2.1	**0.38**

Table 5 shows that decision tree regression gives the least values for RMSE, MAE, and *R*^2^ compared to other regression techniques. At the same time, the Facebook prophet gives the least values for RMSE, MAE, and *R*^2^ compared to other time series models for Mumbai city of Maharashtra. Similarly, Table 6 shows that the random forest model gives the best values for all performance metrics, whereas the AR model gives the least values for RMSE (5.5) and MAE (4.28) for Nagpur city. Table 7 shows that Random forest demonstrates the best performance for metrics RMSE (24.16), MAE (18.88), and *R*^2^ (0.21) for Nashik city. Table 8 shows that random forest gives the best performance for metrics RMSE (14.4), MAE (9.44), *R*^2^ (0.25), and Facebook prophet gives the best performance for metrics RMSE (9.3), MAE (6.7), *R*^2^ (0.64) for Pune city. From all the performance Tables 4–12 and result analysis, it has been observed that Random Forest Regression is the best-fit regression model working on five out of nine cities, i.e., Nagpur, Nashik, Pune, Ratnagiri, Satara, whereas Support Vector Regression shows the best performance on two cities, Thane and Solapur. Facebook Prophet Model is the best fit time series model that worked on six out of nine cities in time series forecasting. For the rest of the cities, various combinations of ARIMA models worked as the best fit.

**Table 5:** Performance metrics comparison table for Mumbai.

**Regression models**	**RMSE**	**MAE**	***R* ^**2**^**	**Time Series forecasting**	**RMSE**	**MAE**	***R* ^**2**^**
Random forest	47.93	34.93	−0.28	Holt's forecasting	127.2	110.9	−2.49
Decision tree	**28.44**	**19.5**	**0.54**	AR	148.5	145.9	−3.76
Support vector	47.55	26.7	−0.26	MA	54.46	50.04	0.35
Multiple linear	60.5	45.25	−1.05	ARIMA	94.62	93.65	−0.93
ElasticNet	55.53	43.11	−0.72	SARIMA	55.7	54.68	0.32
Polynomial	61.01	46.12	−1.08	Facebook prophet	**23.27**	**15.57**	**0.84**

**Table 6:** Performance metrics comparison table for Nagpur.

**Regression models**	**RMSE**	**MAE**	***R* ^**2**^**	**Time series forecasting**	**RMSE**	**MAE**	***R* ^**2**^**
Random forest	**13.56**	**9.72**	**0.12**	Holt's forecasting	7.71	5.95	−0.82
Decision tree	14.89	10.61	−0.05	AR	**5.5**	**4.28**	0.07
Support vector	17.59	10.94	−0.46	MA	7.49	6.09	−0.71
Multiple linear	15.23	11.02	−0.1	ARIMA	5.52	4.43	0.06
ElasticNet	16.17	11.51	−0.24	SARIMA	12.87	11.83	−4.07
Polynomial	29.54	20.57	−3.13	Facebook prophet	9.9	7.28	**0.56**

**Table 7:** Performance metrics comparison table for Nashik.

**Regression models**	**RMSE**	**MAE**	***R* ^**2**^**	**Time series forecasting**	**RMSE**	**MAE**	***R* ^**2**^**
Random forest	**24.16**	**18.88**	**0.21**	Holt's forecasting	19.99	17.69	−3.63
Decision tree	44.55	29.5	−1.68	AR	11.95	10.59	−0.66
Support vector	33.36	21.18	−0.5	MA	15.36	13.33	−1.73
Multiple linear	31.32	24.07	−0.32	ARIMA	**11.72**	10.28	−0.59
ElasticNet	30.2	22.94	−0.23	SARIMA	12.17	**7.86**	−0.72
Polynomial	73.39	50.83	−6.27	Facebook prophet	23.27	16.19	**0.524**

**Table 8:** Performance metrics comparison table for Pune.

**Regression models**	**RMSE**	**MAE**	***R* ^**2**^**	**Time series forecasting**	**RMSE**	**MAE**	***R* ^**2**^**
Random forest	**14.4**	**9.44**	**0.25**	Holt's forecasting	18.25	17.73	−1.3
Decision tree	25.99	18.11	−1.43	AR	21.94	19.63	−2.32
Support vector	20.59	13.28	−0.53	MA	34.07	32.42	−7.02
Multiple linear	14.93	11.09	0.19	ARIMA	25.99	24.24	−3.67
ElasticNet	14.78	10.99	0.21	SARIMA	24.23	21.6	−3.05
Polynomial	18.51	12.21	−0.23	Facebook prophet	**9.3**	**6.7**	**0.64**

**Table 9:** Performance metrics comparison table for Ratnagiri.

**Regression models**	**RMSE**	**MAE**	***R* ^**2**^**	**Time series forecasting**	**RMSE**	**MAE**	***R* ^**2**^**
Random forest	**9.35**	7.31	**0.06**	Holt's forecasting	6.09	5.8	−7.79
Decision tree	15.44	10.16	−1.54	AR	4.42	3.32	−3.62
Support vector	11.46	**7.11**	−0.4	MA	6.57	4.75	−9.24
Multiple linear	11.2	8.96	−0.33	ARIMA	4.49	3.91	−3.79
ElasticNet	10.8	8.47	−0.24	SARIMA	**3.22**	**2.95**	−1.46
Polynomial	18.14	13.12	−2.51	Facebook prophet	7.14	4.89	**0.49**

**Table 10:** Performance metrics comparison table for Satara.

**Regression models**	**RMSE**	**MAE**	***R* ^**2**^**	**Time series forecasting**	**RMSE**	**MAE**	***R* ^**2**^**
Random forest	**5.93**	3.9	**−0.04**	Holt's forecasting	19.99	18.07	−21.21
Decision tree	6.56	4.83	−0.27	AR	13.71	12.92	−9.44
Support vector	6.43	**3.78**	−0.23	MA	13.71	12.92	−9.44
Multiple linear	5.97	4.57	−0.06	ARIMA	13.71	12.92	−9.44
ElasticNet	5.98	4.61	−0.06	SARIMA	12.72	12	−8
Polynomial	16.47	13.11	−7.06	Facebook prophet	**3.98**	3.2	**0.64**

**Table 11:** Performance metrics comparison table for Solapur.

**Regression models**	**RMSE**	**MAE**	***R* ^**2**^**	**Time series forecasting**	**RMSE**	**MAE**	***R* ^**2**^**
Random forest	13.07	9.75	−1.82	Holt's forecasting	18.46	17.66	−12.7
Decision tree	13.38	8.83	−2	AR	21.09	20.51	−16.88
Support vector	**8.2**	**5.14**	**−0.12**	MA	22.85	22.04	−19.99
Multiple linear	15.95	11.77	−3.27	ARIMA	21.09	20.51	−16.88
ElasticNet	15.31	11.34	−2.93	SARIMA	16.94	16.08	−10.53
Polynomial	23.64	18.46	−8.38	Facebook prophet	**7.67**	**5.25**	**0.55**

**Table 12:** Performance metrics comparison table for Thane.

**Regression models**	**RMSE**	**MAE**	***R* ^**2**^**	**Time series forecasting**	**RMSE**	**MAE**	***R* ^**2**^**
Random Forest	34.4	27.77	−0.46	Holt's forecasting	14.65	13.19	−0.61
Decision Tree	35.67	27.16	−0.52	AR	12.88	**10.88**	−0.25
Support Vector	**33.67**	**24.49**	**−0.36**	MA	**12.47**	11.95	−0.17
Multiple Linear	34.12	26.19	−0.39	ARIMA	12.88	**10.88**	−0.25
ElasticNet	37.15	29.27	−0.65	SARIMA	20.13	16.6	−2.05
Polynomial	48.02	37.09	−1.77	Facebook prophet	21.88	15.97	**0.56**

**Figure 7:**
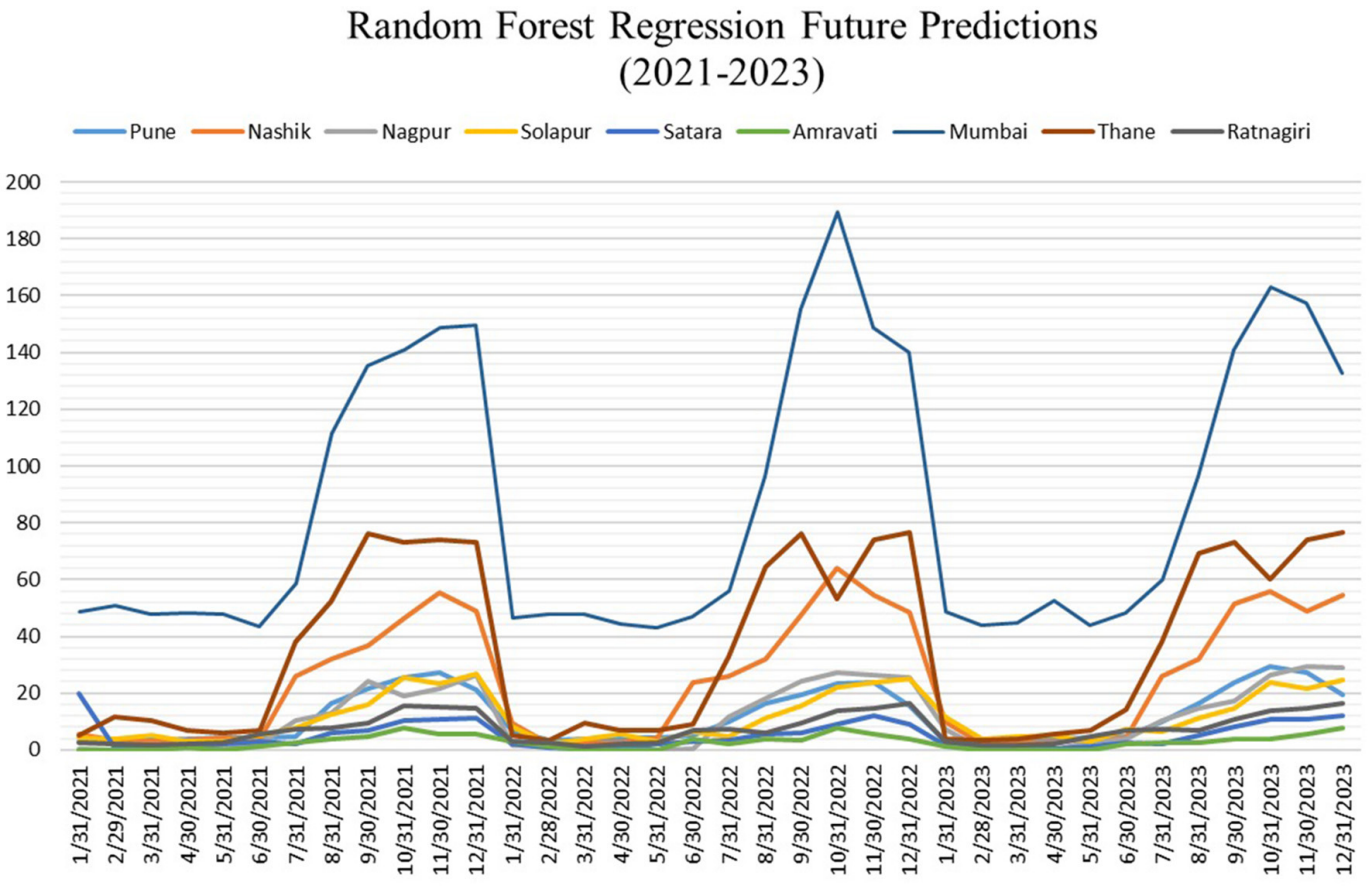
Predictions for random forest regression model (X-axis: Date and Y-axis: Dengue cases).

**Figure 8:**
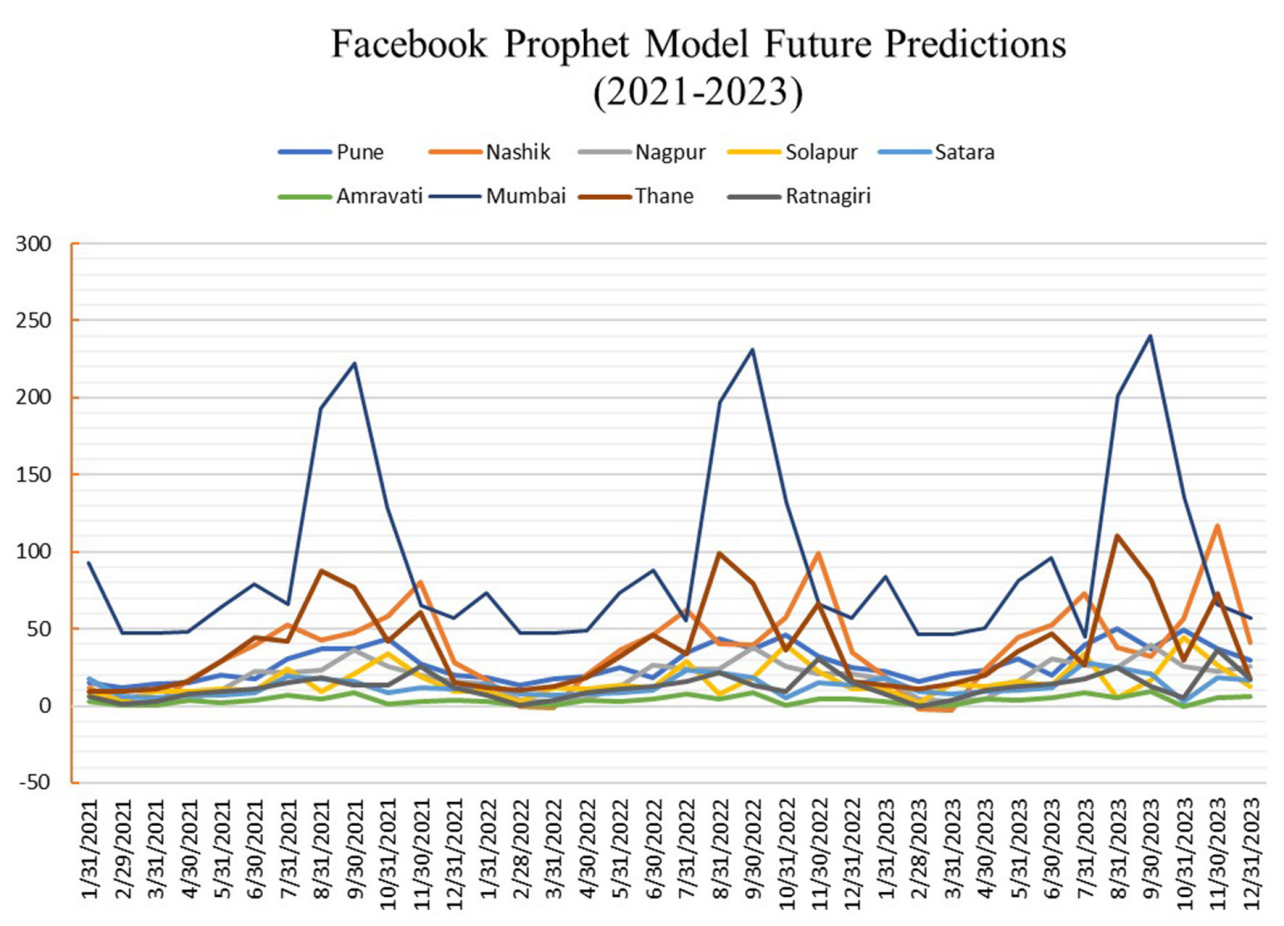
Predictions for Facebook prophet model (X-axis: Date and Y-axis: Dengue cases).

**Figure 9:**
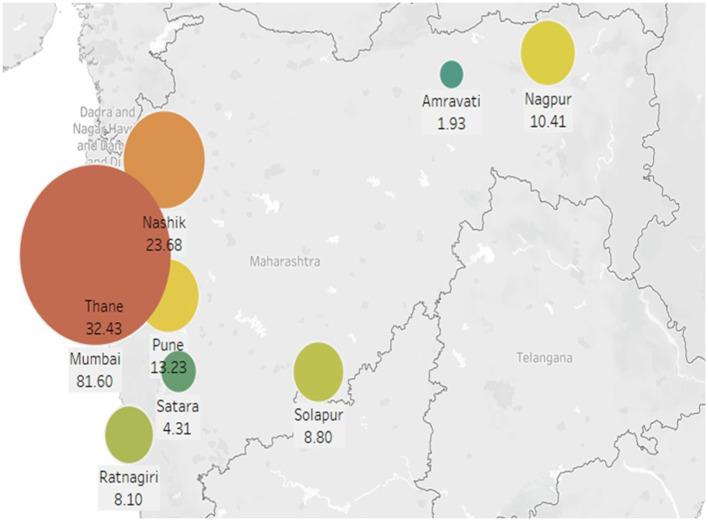
Monthly average incidence hotspot map.

Figure 7 shows predictions for nine targeted cities using Random forest regression, and Figure 8 shows the predictions using the Facebook prophet time series model for 36 months from the Year 2021 to 2023. A hot spot map of Maharashtra state is created, as shown in Figure 9, using Tableau to compare the average number of monthly cases across our selected cities to visualize these results. The figures show that Mumbai is the most affected city, with monthly average dengue cases going up to more than 80, while Amravati is the least affected location of Maharashtra. Other cities are ranged between 5 and 35 cases. Also, Thane, Nashik, and Pune are the cities at high risk, especially in August, September, and October.

## Conclusion, Limitations, and Future Work

### Conclusion

This research paper proposed a framework that can predict dengue incidences across different cities of Maharashtra based on climate parameters. Different meteorological variables like MMIN, MMAX, RH, TMRF, etc., are given as input, and the number of Dengue incidences is produced as output by the proposed system. Nine cities with varied climatic conditions were selected based on geographic regions. A correlation between meteorological parameters and dengue incidences was found out. The proposed system implemented 12 different regression and time series models for the prediction of dengue outbreaks. The performance of all the models is compared using root mean square error, mean absolute error, and R square error evaluation metrics. The result analysis shows that Random Forest outperforms the other Regression models for five out of nine cities. Facebook Prophet Model is the best fit time series forecasting model for six out of nine cities. The system also predicts the high-risk geographic regions from the year 2021 to 2023. It has been observed that Mumbai, Thane, and Pune are the hot spots in Maharashtra, especially from July to October. The medical researchers, public health departments, and health geography analysts can utilize these research results to take the necessary preventive measures based on these predictions.

### Limitations

The study only considers climate factors. Non-climatic factors such as the demography, immunity within the population, society's socio-economic structure, availability of affordable public health facilities, and other environmental modifications initiatives are not considered for the study. Also, there is scope to add additional time-variant factors such as changes in mosquito density, population movements and habits, and vector control measures. The study is limited to a few cities of Maharashtra state of India to analyze monthly climate and dengue incidence data due to the unavailability of weekly or daily reports that could have helped better predictions.

### Future Work

The result of the research will be helpful in designing an effective surveillance system that will effectively monitor and control dengue outbreaks. An output platform like a website can be created to assess the latest climate change parameters, disease outbreaks, and future projections. Future work can involve more extreme geographic regions of India along with daily or weekly climate data analysis. Vulnerability groups such as age, gender, health status, occupation of the patients can be considered to enhance the surveillance system for better planning and preparation to avoid a future outbreak.

## Data Availability Statement

The original contributions presented in the study are included in the article/supplementary material, further inquiries can be directed to the corresponding author.

## Author Contributions

SPat and Span: conceptualization, data collection, interpretation, data curation, methodology, and manuscript writing. All authors contributed to the article and approved the submitted version.

## Funding

The research work has been supported by Symbiosis International (Deemed) University.

## Conflict of Interest

The authors declare that the research was conducted in the absence of any commercial or financial relationships that could be construed as a potential conflict of interest.

## Publisher's Note

All claims expressed in this article are solely those of the authors and do not necessarily represent those of their affiliated organizations, or those of the publisher, the editors and the reviewers. Any product that may be evaluated in this article, or claim that may be made by its manufacturer, is not guaranteed or endorsed by the publisher.
